# Leading the way: finding genes for neurologic disease in dogs using genome-wide mRNA sequencing

**DOI:** 10.1186/1471-2156-13-56

**Published:** 2012-07-10

**Authors:** Elaine A Ostrander, Holly C Beale

**Affiliations:** 1 NHGRI/National Institutes of Health, Building 50, Room 5351, 50 South Drive, Bethesda, MD, 20892, USA

**Keywords:** Dogs, RNAseq, Cortical degeneration, Canines, Neurodegenerative diseases, SPTBN2 gene

## Abstract

Because of dogs' unique population structure, human-like disease biology, and advantageous genomic features, the canine system has risen dramatically in popularity as a tool for discovering disease alleles that have been difficult to find by studying human families or populations. To date, disease studies in dogs have primarily employed either linkage analysis, leveraging the typically large family size, or genome-wide association, which requires only modest-sized case and control groups in dogs. Both have been successful but, like most techniques, each requires a specific combination of time and money, and there are inherent problems associated with each. Here we review the first report of mRNA-Seq in the dog, a study that provides insights into the potential value of applying high-throughput sequencing to the study of genetic diseases in dogs.

Forman and colleagues apply high-throughput sequencing to a single case of canine neonatal cerebellar cortical degeneration. This implementation of whole genome mRNA sequencing, the first reported in dog, is additionally unusual due to the analysis: the data was used not to examine transcript levels or annotate genes, but as a form of target capture that revealed the sequence of transcripts of genes associated with ataxia in humans. This approach entails risks. It would fail if, for example, the relevant transcripts were not sufficiently expressed for genotyping or were not associated with ataxia in humans. But here it pays off handsomely, identifying a single frameshift mutation that segregates with the disease. This work sets the stage for similar studies that take advantage of recent advances in genomics while exploiting the historical background of dog breeds to identify disease-causing mutations.

## Commentary

The domestic dog offers unique advantages to the study of complex and multilocus diseases. Breeds are closed populations; membership requires that all parents and grandparents be registered members of the same breed. The division of the population into over 350 breeds simplifies the locus heterogeneity that is typically associated with complex diseases [1,2]. Finally, many breeds share recent common ancestors [3-5], meaning that they also likely share common disease alleles, offering an avenue to increase power and resolution for genetic studies.

The genome of the dog was sequenced to 7.5x in 2005 [6]. Among the most interesting features are the extensive within-breed linkage disequilibrium (LD) [6,7] and the high degree of across-breed heterogeneity. As a result, genome wide association studies (GWAS) require chips with no more the 100,000 single nucleotide polymorphisms (SNPs) (see [8] for review). Finally, the relatedness of breeds means that transitioning from marker to mutation can often be accomplished by combining data from affected individuals from related breeds [9,10].

Nevertheless, the dog system has some disadvantages. For instance, while dog families may be very large, thus allowing disease genes to be found by linkage analysis of a single large family [11], locating and sampling all the necessary dogs can be problematic. Second, while GWAS have been successful at identifying many loci of interest [12], the extensive LD means that getting from associated locus to mutation can be difficult [8,13].

The recent success of Forman et al, [14] who used genome-wide mRNA sequencing (RNA-Seq) to find the variant associated with a form of neonatal cerebellar cortical degeneration, circumvents many of these problems. By way of background, the disorder is a neurodegenerative disease occurring in several breeds, including the beagle. Affected dogs suffer from a loss of balance, uncoordinated gate, and an inability to regulate movement. Loss of Purkinje cells with swollen dendritic processes is the pathologic hallmark of this recessive disorder [15].

The authors performed genome wide mRNA sequencing using cerebellum tissue from one affected pup. Importantly, they focused their resulting search exclusively on the 27 dog genes that were known orthologs of human ataxia genes. In this sense they got lucky—there are 41 such human loci and causal genes identified for only 28. Of those, dog orthologs are known for 27. Had the causative gene not been previously identified as an ataxia gene in human studies, the cause of the canine disease would not have been found using this approach. After comparison to common dog SNPs and orthologous sequences, variants were eliminated if they were non-coding, heterozygous or conserved. The remaining variant, located in the β-III spectrin gene (*SPTB2),* was an eight base pair (bp) coding deletion that is predicted to cause both an aberrant run of 27 extra amino acids and premature termination of mRNA. The mutation segregated perfectly as an autosomal recessive in the small family tested, was found in the heterozygous state in other unaffected but at-risk dogs, and was absent in 37 other breeds. As expected, cerebellum tissue from the proband, showed a near total loss of both β-III spectrin mRNA and protein when compared to an unspecified control.

β-III spectrin is a superb candidate gene. The gene family encodes cytoskeletal proteins that are important structural components of the plasma membrane. β-III spectrin is found in the nervous system, with the highest levels of expression in the Purkinje cell soma and dendrites [16]. There is, thus, no doubt the correct gene has been found.

While the authors are to be commended for synthesizing a wide range of knowledge and tools to construct a strong argument for their findings, the generalizability of the approach has to be questioned. The authors point out, correctly, that because mRNA-seq requires far fewer samples than does a GWAS, less time is needed to collect samples and complete projects. In this case, however, the authors were well-informed regarding the correct tissue to sequence and candidate genes to consider. That will not always be the case. Also, this approach is likely to miss weakly expressed transcripts, which could be important for some diseases. While not simple, a library normalization step could certainly be incorporated. A major argument for sequencing and pursuing genetic studies in the dog is the simplified genetic architecture: reduced locus heterogeneity improves the chances for identifying variants underlying complex disease traits and a shared genetic background makes disease presentation more uniform. Focusing solely on known human disease genes dismisses both of these advantages.

Does this lessen the impact of the paper? In light of the rapid advances in human genetics, particular with regard to rare genetic diseases, we would argue no. There are many cases where families of human genes are known, but their precise matchup with clinical features remains ambiguous. This practice of using candidate gene/mRNA sequencing will resolve many such cases. The argument for greater clarity in phenotype because of the similar genetic background of breed members can still apply, albeit in a different way. Finally, animal models that result from studies such of that of Forman [14] are clinically valuable.

The number of human genes associate with both common and rare diseases is increasing at an amazing rate. These authors offer a way to take advantage of such advances while making use of the extraordinary advantages of the canine system, resulting in gene discovery that is applicable to both human and companion animal health.

## Conclusions

Applying mRNAseq methods from carefully selected tissues to pre-selected candidate genes, while not a substitute for the GWAS or linkage studies, can reveal causative mutations for multilocus diseases.

## Abbreviations

bp, Base pair; GWAS, Genome wide associations study; LD, Linkage disequilibrium; RNA-seq, mRNA sequencing; SNP, Single nucleotide polymorphism.

## Authors’ contributions

EAO wrote the first draft of the manuscript. HB provided extensive discussion and editing. Both authors contributed to and approved the final version.

## Competing interests

The authors declare no completing interests.
